# Combined *de novo* and genome guided assembly and annotation of the *Pinus patula* juvenile shoot transcriptome

**DOI:** 10.1186/s12864-015-2277-7

**Published:** 2015-12-12

**Authors:** Erik A. Visser, Jill L. Wegrzyn, Emma T. Steenkmap, Alexander A. Myburg, Sanushka Naidoo

**Affiliations:** Department of Genetics, Forestry and Agricultural Biotechnology Institute (FABI), Genomics Research Institute (GRI), University of Pretoria, Private bag X20, Pretoria, 0028 South Africa; Department of Ecology and Evolutionary Biology, University of Connecticut, Storrs, CT 06269 USA; Department of Microbiology and Plant Pathology, Forestry and Agricultural Biotechnology Institute (FABI), Genomics Research Institute (GRI), University of Pretoria, Private bag X20, Pretoria, 0028 South Africa

**Keywords:** *Pinus patula*, *De novo* transcriptome assembly, Genome guided transcriptome assembly, RNA-seq

## Abstract

**Background:**

Pines are the most important tree species to the international forestry industry, covering 42 % of the global industrial forest plantation area. One of the most pressing threats to cultivation of some pine species is the pitch canker fungus, *Fusarium circinatum*, which can have devastating effects in both the field and nursery. Investigation of the *Pinus*-*F. circinatum* host-pathogen interaction is crucial for development of effective disease management strategies. As with many non-model organisms, investigation of host-pathogen interactions in pine species is hampered by limited genomic resources. This was partially alleviated through release of the 22 Gbp *Pinus taeda* v1.01 genome sequence (http://pinegenome.org/pinerefseq/) in 2014. Despite the fact that the fragmented state of the genome may hamper comprehensive transcriptome analysis, it is possible to leverage the inherent redundancy resulting from deep RNA sequencing with Illumina short reads to assemble transcripts in the absence of a completed reference sequence. These data can then be integrated with available genomic data to produce a comprehensive transcriptome resource. The aim of this study was to provide a foundation for gene expression analysis of disease response mechanisms in *Pinus patula* through transcriptome assembly.

**Results:**

Eighteen *d*e *novo* and two reference based assemblies were produced for *P. patula* shoot tissue. For this purpose three transcriptome assemblers, Trinity, Velvet/OASES and SOAPdenovo-Trans, were used to maximise diversity and completeness of assembled transcripts. Redundancy in the assembly was reduced using the EvidentialGene pipeline. The resulting 52 Mb *P. patula* v1.0 shoot transcriptome consists of 52 112 unigenes, 60 % of which could be functionally annotated.

**Conclusions:**

The assembled transcriptome will serve as a major genomic resource for future investigation of *P. patula* and represents the largest gene catalogue produced to date for this species. Furthermore, this assembly can help detect gene-based genetic markers for *P. patula* and the comparative assembly workflow could be applied to generate similar resources for other non-model species.

**Electronic supplementary material:**

The online version of this article (doi:10.1186/s12864-015-2277-7) contains supplementary material, which is available to authorized users.

## Background

*Pinus* species play keystone ecological roles, representing the major component of many forests across all continents [1]. These species are also the predominantly planted trees in the global commercial forestry sector [2]. One of the largest threats to global pine forestry is the pitch canker fungus *Fusarium circinatum*, especially where susceptible *Pinus* species are cultivated [3]*.* Consequent losses caused by this fungus have large economic impacts on commercial forestry [3, 4]. Resistance to *F. circinatum* varies among *Pinus* species [5]. Species such as *Pinus patula* and *P. radiata*, both of which are important plantation species in southern Africa, are highly susceptible, while species such as *P. tecunumanii* are more resistant [5]. Very little is known regarding the interaction between *F. circinatum* and its *Pinus* hosts at the molecular level. Investigation of the different responses employed by susceptible and resistant hosts, such as *P. patula* and *P. tecunumanii* [5], will improve our knowledge of responses necessary for effective defence against *F. circinatum*.

RNA sequencing (RNA-seq) approaches have opened the way for transcriptome-wide analysis of gene expression [6]. Accurate quantification of gene expression using RNA-seq, however, requires a high quality reference sequence for read mapping. For organisms with well characterised reference genomes, such as *Arabidopsis*, this requirement is easily met, while organisms lacking a well characterized reference sequence present numerous challenges. Although the *P. taeda* v1.01 draft genome assembly is available [7], the size and fragmented state of the assembly can limit comprehensive transcriptome analysis [8, 9]. *De novo* transcriptome assembly can be used to provide a reference sequence for RNA-seq analysis while circumventing potential issues arising from problems in a genome assembly [10]. *De novo* transcriptome assemblies are available through GenBank and the TreeGenes database [11, 12] for at least ten *Pinus* species (*P. banksiana* [13]*, P. contorta* [13], *P. lambertiana*, *P. massoniana, P. monticola* [14]*, P. palustris*, *P. pinaster*, *P. radiata*, *P. sylvestris* [15], and *P. taeda*), at various levels of completion. Of these, the *P. taeda* transcriptome is the most comprehensive, consisting of data obtained from many different tissues and developmental stages (Mockaitis et al. unpublished).

The aim of this study was to generate a resource for transcriptome profiling in *P. patula* by assembling the shoot transcriptome of this economically important species. We report a *P. patula* shoot transcriptome containing 52 112 transcripts, of which 30 844 (60 %) are annotated. This is the largest gene catalogue for *P. patula* to date and a major genomic resource, which will facilitate functional genomics research in this tropical pine species.

## Methods

### Plant material

Six month old *P. patula* seedlings, from a single open pollinated family, were sourced from Top Crop Nursery, South Africa. Seedlings were transferred to, and maintained for the duration of the trial in an environmentally controlled glasshouse at 25–28 °C without supplemental lighting and allowed to acclimatize for two weeks. *F. circinatum* isolate FCC3579 was cultured on ½ strength potato dextrose agar (½ PDA; Merck) after which spores were harvested by washing with 15 % (*v/v*) sterile glycerol. Spore concentration was quantified using a haemocytometer and diluted to 5×10^4^ spores/mL by addition of 15 % (*v/v*) sterile glycerol. Seedlings were inoculated by clipping the apical bud and pipetting 10 μL of diluted spore solution onto the wound [16]. Seedlings inoculated with 10 μL sterile 15 % glycerol served as mock-inoculated control. Shoot tissue was harvested one day post inoculation (dpi) for three biological replicates per group (inoculated and mock-inoculated). Each biological replicate consisted of the top 4 cm of shoot tissue, measured from the wounded apical bud, harvested from 16 seedlings and pooled prior to being frozen using liquid nitrogen. Frozen tissue was stored at −80 °C until use. Disease development was monitored for six weeks post inoculation by measuring lesion and stem length from the wounded apical bud on 52 plants per group. *F. circinatum* infection was confirmed based on culture morphology on ½ PDA by re-isolation using tissue harvested from inoculated plants 14 dpi.

### RNA isolation and sequencing

Frozen samples were homogenised using a high speed grinder (IKA-Werke, Staufen, Germany) and total RNA extracted using a modified version of Lewinsohn’s protocol [17]. Modifications were as follows: All solutions were prepared using diethylpyrolecarbonate (DEPC) treated water. Approximately 5 g homogenised shoot tissue was placed in a 50 mL conical tube containing 150 mg PVP-360 and 300 mg PVPP before adding 15 mL chilled extraction buffer. Tubes were mixed by vortexing, snap frozen in liquid nitrogen and allowed to thaw on ice. Polysaccharides were precipitated by addition of 1/9^th^ volume 3.3 M sodium acetate and 10 % (*v/v*) absolute ethanol. Nucleic acids were precipitated at −20 °C for 4 h. The pellet produced from ultracentrifugation was re-suspended in 100 μL DEPC treated water and stored at −80 °C until use. Total RNA samples were treated with RNase-free DNaseI enzyme (Qiagen Inc, Valencia, CA) to digest genomic DNA and purified using the RNeasy® MinElute kit (Qiagen) according to the manufacturer’s instructions. Concentration and integrity of purified RNA samples were evaluated using a Bio-Rad Experion™ automated electrophoresis system (Bio-Rad Laboratories, Hercules, CA, USA).

High quality RNA samples (RNA Integrity Number > 7.5) for both groups were sequenced using Illumina HighSeq 2000 instruments (200 bp insert size, 101PE sequencing, 40 million reads per sample; BGI, Hong Kong). Sequence quality of raw RNA-seq data was assessed using FastQC v0.10.1 [18]. Quality trimming and filtering of data was performed using Sickle v1.210 [19] and all unpaired reads were discarded. Short reads (<40 bp) were removed from the filtered RNA-seq reads using SolexaQA LengthSort [20]. The trimmed and filtered read data for all six samples were combined, resulting in Dataset 1. FastUniq v1.1 [21] was used to reduce PCR artefacts from Dataset 1 by removing duplicate reads, resulting in Dataset 2.

## Transcriptome assembly

### Multiple *k*-mer *de novo* transcriptome assembly

*De novo* transcriptome assembly was performed using three assemblers; Trinity r2013-11-10 [22], SOAPdenovo-Trans v1.04 [23] and Velvet v1.2.10/ Oases v0.2.08 [24, 25]. Assembly with Trinity was performed on both datasets using default parameters [26], except min_contig_length = 350, and repeated on Dataset 1 with the CuffFly parameter included. Trinity was applied to both Dataset 1 and 2 as Trinity allows for duplicate reads, however, SOAPdenovo-Trans and Velvet/Oases assemblers were used on Dataset 2 only, where duplicates were removed. Assembly with SOAPdenovo-Trans was performed on Dataset 2, for eight different *k*-mer lengths (23, 25, 31, 39, 47, 55, 63 and 71), with the parameters as follows: max_rd_len = 100, rd_len_cutoff = 100, avg_ins = 200, reverse_seq = 0, asm_flags = 3, pair_num_cutoff = 3, map_len = 35, −f and -F. Assembly with Velvet/Oases was performed on Dataset 2, for seven different *k*-mer lengths (23, 25, 31, 39, 47, 55, and 63), with the parameters as follows: default parameters for velveth; cov_cutoff = 10, ins_length = 200 and read_trkg = yes for velvetg; cov_cutoff = 10, min_pair_count = 5 and min_trans_lgth = 350 for Oases.

### *P. taeda* v1.01 genome guided transcriptome assembly

Trinity genome guided transcriptome assembly was performed on Datasets 1 and 2 using the *P. taeda* v1.01 draft genome assembly (*ca.* 14.4 million scaffolds) with a minimum contig length of 350 bp. GSNAP 2014-02-28 (Genomic Short-read Nucleotide Alignment Program) [27] was used to align reads to the reference genome for transcriptome assembly using the following paramters: −-nofails, −-novelsplicing = 1, −-localsplicedist = 250000, −-npaths = 20. Transrate v0.3.1 [27] was used to calculate assembly quality metrics.

### Decreasing redundancy across assemblies

The *de novo* and genome guided transcriptome assemblies were combined to form a redundant over-assembly. The tr2aacds pipeline, from the EvidentialGene package [28], was used to reduce redundancy in the over-assembly by selecting for the ‘optimal’ set of assembled transcripts based on coding potential. The pipeline consists of five steps: (1) prediction of coding DNA sequence (CDS) and amino acid sequences for each transcript, (2) removal of redundant sequences based on coding potential among identical sequences, (3) substring de-replication to remove sequence fragments, (4) clustering of highly similar sequences into loci and (5) classification of transcripts as ‘primary’ or ‘alternate’ and discarding of low scoring ‘drop’ transcripts. The primary assembled transcripts were used for further assessments.

### Annotation

Local alignments to the National Centre for Biotechnology Information (NCBI) non-redundant (nr) and plant protein databases were generated for the primary assembled transcripts from the tr2aacds pipeline using uBLAST (Edgar RC, *unpublished*) [29]. Parameters used for local alignments were: −evalue 1e-10, −weak_evalue 1e-4, −id 0.9, −weak_id 0.8. Local alignment sequence descriptions were used to remove non-pine origin sequences, sequences with significant alignments to prevalent fungal, bacterial, viral and insect sequences, from the assembly to produce the *P. patula* v1.0 draft transcriptome assembly. Blast2GO® v2.7.2 [30] was used to predict protein domains through InterProScan 5 [31] as well as to perform Gene Ontology (GO) and Enzyme Code (EC) mapping. The *P. patula* transcriptome GO distribution was compared to the *P. taeda* v1.01 draft genome annotation using CateGOrizer [32]. Gene family memberships among species were visualized using custom scripts and Venn diagrams (http://bioinformatics.psb.ugent.be/webtools/Venn/).

### Identification of orthologous protein groups

Annotated protein sequences for ten different species were retrieved from version 2.5 of the PLAZA protein database [33] and four external proteins sets, from conifer species, were also included (Table 1). The complete set of predicted *P. patula* v1.0 proteins from the assembled transcriptome were included. Each of the 15 protein sets were clustered to 90 % identity within species and combined. Gene families were identified and annotated for the 442 372 sequences using the approach described in [8]. Pfam domains [34] were assigned to the *P. patula* sequences using InterProScan 5.7 [31]. Identified gene families unique to *P. patula* with fewer than 5 members were discarded as these could result from assembly artefacts.

**Table 1:** Protein sets used for analysis of orthologous genes

Source	Species	Total sequences	Clustered sequences^a^
Protein sets from PLAZA v2.5	*Arabidopsis thaliana*	27 403	26 465
	*Glycine max*	46 324	36 364
	*Oryza sativa*	41 363	39 541
	*Physcomitrella patens*	28 090	26 072
	*Populus trichocarpa*	40 141	35 668
	*Ricins communis*	31 009	30 330
	*Selaginella moellendorffii*	18 384	16 876
	*Theobroma cacao*	28 858	28 294
	*Vitis vinifera*	26 238	24 635
	*Zea mays*	39 172	34 664
External protein sets	*Amborella trichopoda*	25 347	24 643
	*Picea abies*	22 070	20 869
	*Picea sitchensis*	10 521	8 770
	*Pinus patula*	52 112	41 956
	*Pinus taeda*	50 172	47 225

^a^Proteins were clustered to 90 % identity and only the longest sequence was retained for each cluster

### Assembly validation

The Core Eukaryotic Genes Mapping Approach (CEGMA) pipeline [35] as well as the Benchmarking Universal Single-Copy Orthologs (BUSCO) v1.1b1 tool [36] were used to identify putative core eukaryotic genes (CEGs) and universal single copy orthologs (USCOs) in the assembly as a measure of the completeness and contiguity. BUSCO analysis was performed using the early access plant dataset. In addition, conditional reciprocal best BLAST (CRBB) analysis of the *P. patula* draft transcriptome assembly, the *P. taeda* v1.01 gene models and the *P. taeda* draft transcriptome assembly was implemented with two different sets of reference sequences using Transrate [27]. Reference sets used were as follows: the *P. taeda* v1.01 predicted gene models available through the TreeGenes Database [11, 12] and the 87 *P. patula* protein sequences available through the NCBI and TrEMBL databases.

Sequence alignments against the *P. taeda* v1.01 draft genome assembly were generated to compare transcript to genome mapping of the *P. patula* v1.0 transcriptome assembly to that of other *Pinus* transcriptomes. Comparative alignments were produced using transcriptome data for seven other *Pinus* spp. available from the TreeGenes database [11, 12]: *P. taeda* (83 285 sequences), *P. banksiana* (21 675), *P. contorta* (14 375), *P. pinaster* (14 130), *P. palustris* (14 228), *P. lambertiana* (48 891), and *P. radiata* (4 742). Transcript sequences were aligned to the reference genome using GMAP 2014–02-28 (Genomic Mapping and Alignment Program) [37] with the following parameters: −-intronlength = 350000, −-no-chimeras, −-canonical-mode = 1, −-cross-species. The ‘--cross-species’ parameter was excluded for alignment of the *P. taeda* transcriptome. Sequence alignments were examined at two different cut-offs, the first (95 % identity, 95 % coverage) to compare mapping between species and the second (95 % identity, 50 % coverage) to account for possible effects due to genome fragmentation. The *P. patula* v1.0 transcriptome assembly was further validated by alignment to full-length Sanger sequenced *P. taeda* cDNA reference sequences present in NCBI and obtained through the TreeGenes database [11]. The 188 cDNA sequences were clustered to 90 % identity. CRBB analysis to the *P. patula* v1.0 transcriptome was performed using Transrate [27].

### Differential expression analysis

RNA-seq read mapping to the *P. patula* v1.0 transcriptome and expression quantification was performed through RSEM v1.2.23 (RNA-Seq by Expectation-Maximum) [38] using Bowtie2 v2.2.5 [39]. Differential expression testing was performed with EBSeq v1.10.0 [40] using median normalization (FDR < 0.05).

## Results and discussion

### Data generation and pre-processing

Due to the expected size of the *P. patula* genome (*ca.* 22 Gb) [41], sequencing and assembly of the genome would be a costly and challenging endeavour. Therefore, transcriptome assembly was employed to generate a *P. patula* reference sequence. RNA-seq of shoot tissue harvested 1 dpi for inoculated and mock-inoculated samples yielded between 21 and 43 million read pairs per sample and a total of *ca.* 440 million reads (Table 2). Quality filtering removed *ca.* 13 % of reads and duplicate filtering removed a further 35 % of reads. Thus Dataset 1 consisted of *ca.* 36 Gb of sequence data and Dataset 2 consisted of *ca.* 23 Gb that passed through quality filtering and were subsequently used for transcriptome assembly.

**Table 2:** Quality statistics for RNA sequencing data

Data Set	Sample Name	Total Reads	Length (nt)	Q30^a^ (%)	Total (Gb)
Raw Data	Mock-inoculated 1	78 344 666	100	87.1	7.83
	Mock-Inoculated 2	86 178 906	100	87.1	8.62
	Mock-Inoculated 3	82 271 364	100	87.1	8.23
	Inoculated 1	41 756 756	100	86.8	4.18
	Inoculated 2	71 697 894	100	87.1	7.17
	Inoculated 3	80 588 142	100	87.1	8.06
	Total	440 837 728			44.08
Pre-processed Data	Mock-inoculated 1	68 237 135	41–100	100.0	6.45
	Mock-Inoculated 2	75 081 850	41–100	100.0	7.10
	Mock-Inoculated 3	71 683 022	41–100	100.0	6.77
	Inoculated 1	36 270 688	41–100	100.0	3.43
	Inoculated 2	62 484 245	41–100	100.0	5.90
	Inoculated 3	70 235 351	41–100	100.0	6.64
Dataset 1	Total	383 992 290	41–100	100.0	36.29
Dataset 2	Total	248 994 870	41–100	100.0	23.53

^a^Percentage of reads in the library with a Phred score > 30

### Comparison of assembler output

**Fig. 1:**
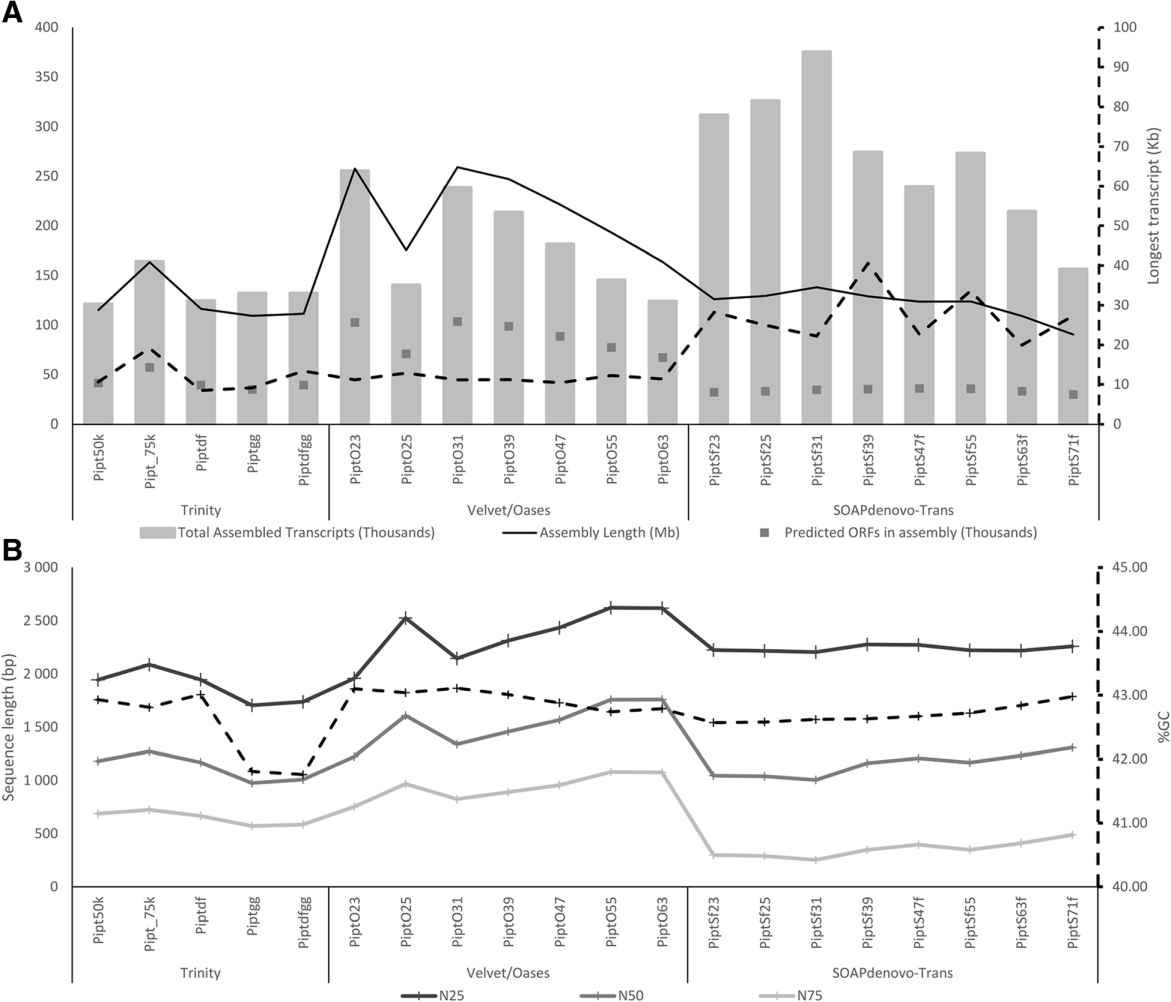
Summarised assembly statistics for all preliminary assemblies. Pipt = *Pinus patula* (**a**) – Assembly size and length statistics. (**b**) –Transcript N statistics and GC ratio for all assemblies. In each case the right hand y-axis only applies to the dashed line. The first three Trinity assemblies were *de novo* assemblies using Dataset 1 with (50 k) and without (75 k) CuffFly, and using Dataset 2 (df). The last two Trinity assemblies represent reference guided assemblies using Dataset 1 (gg) and Dataset 2 (dfgg). For Velvet/Oases and SOAPdenovo-Trans, the numbers indicated the *k-*mer value used. ORF = open reading frame

The completeness and quality of an assembled transcriptome is affected by the assembly program used as well as the assembly parameters used [42–48]. Comparative studies have also shown that the effectiveness of assembly programs can vary by input data set, with no assembler consistently outperforming any other [42, 43]. Due to this variability among assembler outputs, each variant assembly is likely to contain more accurate and complete assemblies at different loci. Therefore, in an effort to maximise diversity of assembled transcripts, we produced 18 *de novo* and two genome guided transcriptome assemblies using; Trinity, SOAPdenovo-Trans and Velvet/Oases. As expected from previous studies, large variation in the number, length and redundancy of contigs assembled was observed within and between assemblers (Fig. 1).

Trinity exhibited the most uniformity among assemblies compared to the variation among assemblies from Velvet/Oases and SOAPdenovo-Trans. An inverse relationship has been shown to exist between the number of contigs assembled and *k-*mer length used for assembly [43]. Therefore, the greater uniformity in assembled contig number between Trinity assemblies can likely be attributed to the program’s implementation of a fixed *k*-mer length for all assemblies. For each value of *k* used in assembly, SOAPdenovo-Trans resulted in the highest number of assembled contigs followed by Velvet/Oases and lastly, Trinity. In a comparative study, Trinity consistently assembled more contigs than Velvet/Oases and Trinity assemblies consistently had a higher N50 statistic [45]. Although the present study used newer versions of Velvet/Oases and Trinity, the difference in trends obtained illustrates the difference in performance of assemblers under different conditions, supporting the need for use of multiple assemblers during transcriptome reconstruction.

Trinity genome guided assemblies displayed lower GC ratios, as well as fewer predicted open reading frames (ORFs) compared to other assemblies (Fig. 1). This was ascribed to fragmentation of the *P. taeda* v1.01 genome. Nevertheless, the two genome guided Trinity assemblies were included in downstream analysis. In total, 3 447 807 assembled transcripts were used as input for the EvidentialGene tr2aacds pipeline.

**Fig. 2:**
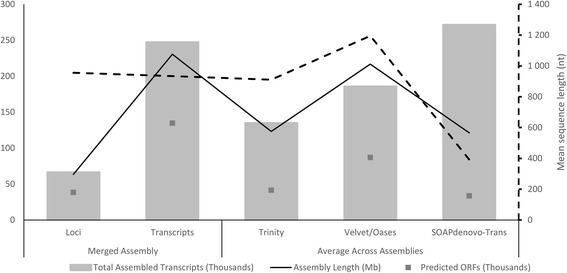
Assembly statistics for tr2aacds pipeline merged assembly compared to average assembly statistics for each assembler. Assembly size and length statistics. The dashed y-axis only applies to the dashed line. Unfiltered output assemblies from Trinity, Velvet/Oases and SOAPdenovo-Trans were used

The EvidentialGene pipeline selects the ‘best’ transcripts based on coding potential, thus selecting for the best ORFs assembled. Open reading frames were successfully predicted for *ca.* 2.7 million (77 %) of the input transcripts. Of these, 49 % were classified as redundant and 51 % were classified as differing in CDS (non-redundant). A further 55 % of non-redundant sequences were classified as perfect fragments of other longer CDS, leaving 23 % of the predicted 2.7 million CDS as informative. Of the informative CDS, 60 % were assigned to the ‘drop’ category and discarded. Overall, this brought about a 14-fold reduction in assembled transcript number, with only 7 % of the original input sequences maintained. The resulting merged assembly contained 247 035 transcripts grouped into 66 377 predicted loci (Additional file 1: Table S1). This assembly was compared to the average assembly statistics across assemblies for each assembly program respectively (Fig. 2; Additional file 2: Table S2). Despite the decrease in transcript number, the proportion of transcripts containing a predicted ORF in the merged assembly was 10–40 % higher compared to the average ORFs per assembly for all three assemblers. This indicates that a higher proportion of transcripts in the merged set have been accurately assembled to near-full or full length. The average length among the 1 000 longest predicted proteins in the merged assembly was 1 425 amino acids. Due to the high resource expenditure required to produce these long proteins they are often well-conserved and a biological maximum has been observed for their average length [28]. For plants this maximum observed average is *ca.* 1 500 amino acids (based on the average between Arabidopsis, banana, cacao and poplar) [28]. This indicates that these proteins are well assembled in the merged assembly, although there is room for improvement.

### Annotation

Sequence homology searches against the NR and plant protein databases successfully obtained significant local alignments for 32 416 loci. Of these, 14 255 were classified as non-pine origin transcripts and filtered from the transcriptome assembly. The majority of non-pine origin transcripts (71.62 %) aligned to transcripts from *Fusarium* species (Additional file 3: Table S3), with most aligning to either *F. fujikuroi* (4 698 transcripts) or *F. oxysporum* (3 290 transcripts). A further 22.81 % of non-pine origin transcripts aligned to *Bipolaris maydis* (1 309 transcripts), *Pyrenophora tritici-repentis* (992 transcripts) and *Leptosphaeria maculans* (949 transcripts), while the remaining 5.57 % (798 transcripts) aligned to 146 different species.

Removal of non-pine origin transcripts resulted in 52 112 putative loci, classified as the *P. patula* v1.0 shoot transcriptome. The current estimates for conifer gene numbers lie between *ca.* 32 000 for *Picea glauca* [49] and *ca.* 50 000 for *P. taeda* and *P. pinaster* [8, 50]. Roughly 60 % of assembled *P. patula* transcript sequences were successfully annotated, representing a wide array of molecular functions, biological processes and cellular compartment GO terms. The remaining 40 % of the assembled transcript sequences could not be annotated through similarity searches, however, each sequence contained an ORF predicted by the EvidentialGene pipeline and could potentially be expressed. Thus, these sequences were not removed from the assembly as they could represent uncharacterised or conifer specific genes. The top molecular function terms for *P. patula* v1.0 transcriptome were protein binding, transferase activity and nucleic acid binding (Additional file 4: Figure S1), similar to what has been observed for *P. taeda*, *P. glauca* and *Picea mariana* [8, 51].

### Identification of putative NB-ARC defence related gene families

**Fig. 3:**
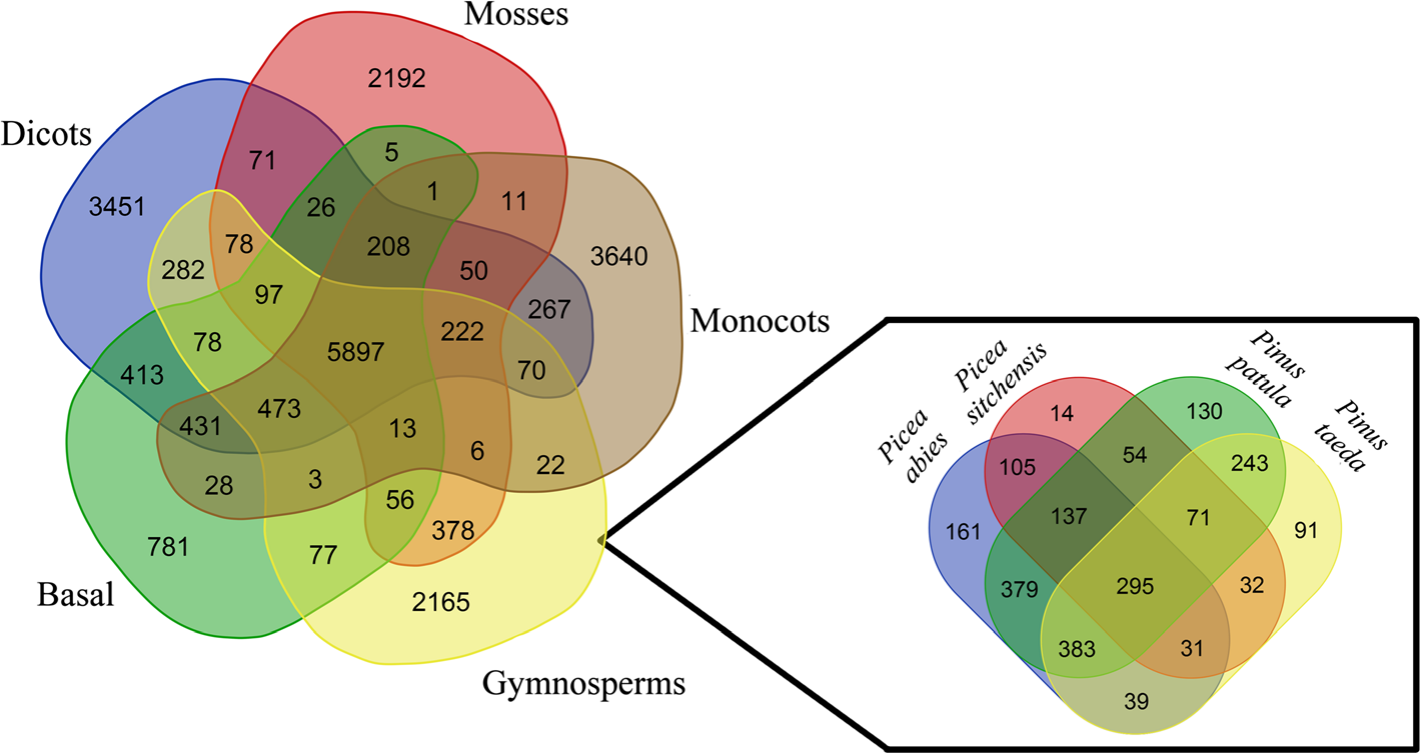
Unique orthologous protein groups identified through Tribe-MCL analysis. *Left* Comparison of protein family counts for all identified orthologous protein groups between five different plant classifications. *Right* Comparison of conifer specific protein counts between four conifer species. Dicots = *Arabidopsis thaliana*, *Glycine max*, *Populus trichocarpa*, *Ricinus communis*, *Theobroma cacao*, *Vitis vinifera*. Mosses = *Selaginella moellendorffii*, *Physcomitrella patens*. Monocots = *Oryza sativa*, *Zea mays*. Gymnosperms = *Picea abies*, *Picea sitchensis*, *Pinus patula*, *Pinus taeda*. Basal = *Amborella trichopoda*

**Fig. 4:**
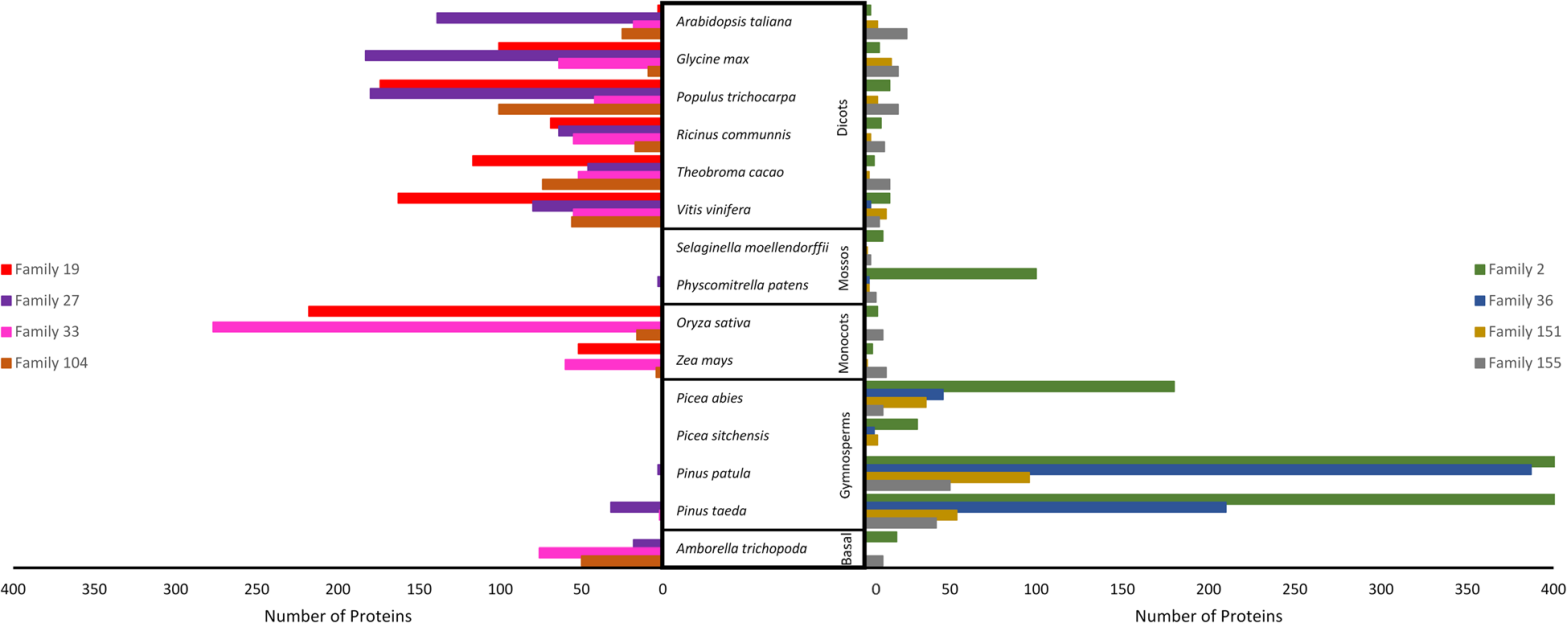
Number of proteins per species for the eight most populated NB-ARC motif containing gene families identified. Gene families were identified using Tribe-MCL. *Left* – NB-ARC families most prominent in conifers. *Right* – NB-ARC families most prominent in angiosperms. Each color represents a different gene family (Additional file 2: Table S2). Family 2 (*green bars*) for *P. taeda* and *P. patula* had 852 and 1 794 members respectively

Orthologous protein groups were identified by comparing 41 956 clustered *P. patula* protein sequences from the assembled transcriptome to the 400 416 clustered protein sequences from 14 other plant species [52] (Table 1). Tribe-MCL analysis [53] resulted in 21,492 unique gene families, with an average of 18 members per family (Additional file 5: Table S4). Gene families were identified for 396 684 (89.6 %) sequences and ranged in size from 6 258 members from 15 species to 2 members from one species. Genes from the *P. patula* v1.0 transcriptome assembly initially clustered to 9 677 gene families (35 433 genes). This was reduced to 8 743 families (33 367 genes) by removing *P. patula* specific gene families with less than 5 members. While there are likely valid families in the removed set, these families were removed as most are likely to have arisen due to the remaining heterozygosity in the assembly. Of the total gene families, 2 165 were unique to conifers (Fig. 3). Although this is higher than the 1 554 reported by the *P. taeda* genome project [7, 8], it is a similar increase from the 1 021 reported by the *P. abies* genome project [54]. Included in the conifer-specific gene families are 130 that were unique to *P. patula*. The largest family identified in *P. patula* (1 794 members) contained leucine rich repeat (LRR), toll/interleukin-1 receptor (TIR), nucleotide binding domain with an ARC motif (NB-ARC), golgi transport complex 5 (COG5) and poxvirus A32 protein motifs. This gene family was also one of the largest observed for *P. taeda* and had low representation among the angiosperms while representation in the moss species differed. In total, 35 NB-ARC families were identified, of which 13 were present in conifers. NB-ARC gene families with higher representation of angiosperm genes had little to no representation from the conifers and vice versa (Fig. 4). The NB-ARC family of genes are associated with disease resistance as the majority of resistance proteins (R proteins) characterized are members of the NB-ARC and NB-LRR families [55]. Thus this difference could result from divergent R gene evolution between the plant lineages.

### Assembly validation

Many well established metrics exist for assessment of genome assembly quality, the majority of which are based on size, such as contig and N50 size. Size based metrics such as N50 have been used in the past as a measure of transcriptome completeness [56], yet these metrics have no real biological relevance and are ineffective without prior knowledge of the actual size distribution in the sequenced data set. These metrics are also highly sensitive to assembly parameters and assembled isoforms (Fig. 1), which can bias quality assessment. For this reason, three reference based metrics were used to assess the transcriptome assembly; completeness, contiguity and accuracy [10]. Completeness and contiguity are closely linked. Completeness is the percentage of a reference set that has been assembled. Contiguity is the percentage of assembled reference sequences covered to X%, where X is an arbitrary minimum threshold [56]. In this study, contiguity and completeness of the *P. patula* transcriptome assembly was measured by comparison against four data sets.

Comparison to the CEGMA core eukaryotic proteins identified 217 (88 % completeness) of the 248 core genes, whereas 203 (82 % completeness) were identified in the *P. taeda* v1.01 genome [7]. At the same time, of the identified core genes, 91 % showed full length alignments to the *P. taeda* v1.01 genome, while 93 % (contiguity) of those from the *P. patula* v1.0 shoot transcriptome were full length. The higher completeness and contiguity obtained for CEGs in the *P. patula* transcriptome assembly compared to the *P. taeda* v1.01 genome can most likely be attributed to genome fragmentation. This illustrates the value of *de novo* transcriptome assembly for analysing genes missing from an incomplete genome. CEGMA analysis also identified multiple orthologs for 90 % of the identified CEGs. This is likely due to the presence of high allelic variation in the data used for assembly arising from the pooled nature of the samples (pooled RNA from seedlings) used for sequencing.

BUSCO analysis against the early access plant data set identified 850 (88 %) complete BUSCOs, out of 956 groups searched, of which 307 (32 %) were duplicated. A further 26 fragmented BUSCOs were identified. The high amount of duplicated complete BUSCOs further indicate the presence of assembled haplotypes still present in the transcriptome [36].

CRBB analysis to the *P. patula* reference proteins showed a similar pattern as above when comparing completeness (49 %) and contiguity (92 %) of the *P. patula* transcriptome to that of the *P. taeda* transcriptome (43; 92 %). This indicates higher completeness of the *P. patula* transcriptome for *P. patula* origin proteins as would be expected. The completeness (46 %) of the *P. taeda* gene models was intermediate between the transcriptomes, however, its contiguity (13 %) was notably lower. This low contiguity most likely arose due to the presence of partial genes in the high confidence gene models. The completeness and contiguity of the *P. patula* transcriptome assembly was also investigated through CRBB analysis to the *P. taeda* v1.0 transcriptome assembly using the gene models extracted from the *P. taeda* v1.01 genome assembly and the 87 available *P. patula* protein sequences as the reference sets. Compared to the *P. taeda* transcriptome, the *P. patula* transcriptome covered a higher proportion of the *P. taeda* gene models (at 95 % coverage), had a higher proportion of reference sequences with a CRBB result and had the lowest reciprocal best hit (RBH) ratio (Table 3). Overall CRBB statistics for comparison to the *P. patula* proteins were higher for the *P. patula* transcriptome compared to both the *P. taeda* transcriptome and gene models (Table 3).

**Table 3:** Conditional reciprocal best BLAST (CRBB) comparisons^a^ of assembled *Pinus patula* transcripts to available *Pinus taeda* gene models and transcripts

Query	*P. taeda* gene models	*P. taeda* v1.0	*P. patula* v1.0
Reference	*P. taeda* gene models (*n* = 48 391)		
Hits at 85 % coverage	99.7 %	6.6 %	8.3 %
Hits at 95 % coverage	99.7 %	3.7 %	4.2 %
Contigs with CRBB	48 363	29 052	28 491
% Contigs with CRBB	99.9 %	34.9 %	54.7 %
References with CRBB	48 269	12 339	15 958
% Reference CRBB	99.7 %	25.5 %	33.0 %
Reciprocal Best Hit Ratio	1.00	2.35	1.79
Reference	*P. patula* proteins (*n* = 87)		
Hits at 85 % coverage	5.8 %	39.1 %	43.7 %
Hits at 95 % coverage	2.3 %	34.5 %	40.2 %
Contigs with CRBB	73	80	71
% Contigs with CRBB	0.2 %	0.1 %	0.1 %
References with CRBB	40	37	43
% Reference CRBB	46.0 %	42.5 %	49.4 %
Reciprocal Best Hit Ratio	1.83	2.16	1.65

^**a**^CRBB alignments for query sequences were generated against the available high confidence *P. taeda* gene models and the available *P. patula* protein sequences

The third metric assessed was accuracy, defined as the percentage of correctly assembled bases in an assembly compared to a reference [10]. This was estimated through high-identity mapping of the assembled *P. patula* transcriptome, along with seven other pine transcriptomes, to the *P. taeda* v1.01 genome (Table 4). Mapping to the genome precluded calculation of completeness and contiguity, due to genome fragmentation and lack of exact gene number and location. At 95 % sequence identity and query coverage thresholds a total of 64 % of *P. patula* sequences mapped. The highest total mapping rates were observed for *P. banksiana* and *P. contorta*, while the lowest mapping rate was obtained for *P. lambertiana*, as expected from their phylogenetic relationship and previous studies [8]. Mapping rates obtained for the *P. patula* transcriptome were similar to the mapping rates obtained for the *P. radiata* and *P. taeda* transcriptome assemblies. These alignment metrics serve as a measure of transcriptome accuracy. Lowering the minimum coverage threshold to 50 % increased mapping by between 2 % and 15 %. The *P. banksiana* (3.8 %), *P. contorta* (2.5 %) and *P. patula* (2.8 %) transcriptomes were the least affected, while the transcriptomes for *P. radiata* (15.1 %), *P. sylvestris* (10.3 %) and *P. taeda* (13.1 %) showed the largest increase in mapping rates, suggesting that these transcriptomes have a higher content of genes that were fragmented in the genome assembly. Comparison of accuracy metrics between assemblies should be done with care, however, as even though the *P. taeda* transcriptome showed a lower accuracy (57 %) than *P. patula*, the size of the transcriptome means that it still contains *ca.* 10 000 more accurately assembled sequences. This illustrates the importance of considering assembly size when comparing between datasets, such as the high mapping rates to the *P. taeda* v1.01 genome obtained for *P. contorta*, *P. pinaster* and *P. radiata* (Table 4). Still, more than 33 000 (64 %) of the assembled *P. patula* sequences were shown to be accurately assembled and this number is expected to increase as fragmentation in the genome decreases.

**Table 4:** Mapping statistics to the *P. taeda* v1.01 genome

Assembly	Total Sequences	Identity	Coverage	Unique Hits	Non-unique hits	Total % mapped
*Pinus patula *(EviGene Loci)	66 377	95	95	33.53 %	16.78 %	50.31 %
	66 377	95	50	34.44 %	18.10 %	52.55 %
*Pinus patula *v1.0	52 112	95	95	42.68 %	21.35 %	64.03 %
	52 112	95	50	43.84 %	23.04 %	66.87 %
*Pinus banksiana*	21 675	95	95	73.25 %	15.26 %	88.51 %
	21 675	95	50	74.72 %	17.62 %	92.34 %
*Pinus contorta*	14 375	95	95	70.23 %	14.91 %	85.15 %
	14 375	95	50	70.37 %	17.27 %	87.64 %
*Pinus lambertiana*	48 891	95	95	25.16 %	1.07 %	26.23 %
	48 891	95	50	31.04 %	2.07 %	33.11 %
*Pinus pinaster*	14 130	95	95	56.24 %	12.76 %	69.00 %
	14 130	95	50	61.27 %	16.28 %	77.54 %
*Pinus radiata*	4 742	95	95	46.06 %	11.66 %	57.72 %
	4 742	95	50	57.11 %	15.67 %	72.78 %
*Pinus sylvestris*	11 248	95	95	47.75 %	16.79 %	64.54 %
	11 248	95	50	53.90 %	20.96 %	74.87 %
*Pinus taeda*	83 285	95	95	48.72 %	7.82 %	56.54 %
	83 285	95	50	57.64 %	11.99 %	69.63 %

The assembled *P. patula* transcripts were further compared to corresponding *P. taeda* complete CDS sequences to ascertain the quality of the assembly against experimentally validated data (Additional file 6: Table S5). Of the 121 cDNA sequences, 89 (73.5 %) mapped to the *P. patula* transcriptome with greater than 89 % identity and 80 % subject coverage (Additional file 6: Table S5). Of the mapped sequences, 47 had a query coverage of more than 80 % with an average sequence identity of 98.4 ± 1.9 % and an average coverage of 97.9 ± 3.5 % and 91.5 ± 6.8 % for the subject and query sequences respectively (Additional file 7: Figure S2). Thus, of the *P. taeda* cDNA sequences present in the assembled *P. patula* transcriptome, 52.8 % have been assembled to near full-length.

### Differential expression analysis

Comparison of inoculated and mock-inoculated data sets using EBSeq identified 166 transcripts as differentially expressed between conditions (Additional file 8: Table S6). The small number of detectable differentially expressed transcripts is likely a reflection of the very early time-point investigated, where small amounts of pathogen would have been in contact with the host tissue.

Ten transcripts were up-regulated (log_2_(fold change) > 1) in the inoculated set, relative to mock-inoculated, while 156 transcripts were down-regulated (log_2_(fold change) < −0.25; 77 had log_2_(fold change) < −1). Among the up-regulated genes four had putative annotations (Additional file 9: Table S7). Two of these genes are involved in folate metabolism (methylenetetrahydrofolate dehydrogenase) and stomatal closure (PF03595), while the other two are linked to sugar metabolism (PREDICTED: alpha-galactosidase-like; sucrose synthase-like protein).

In the down-regulated set 83 transcripts had putative annotations (Additional file 9: Table S7). Some of these are related to plant defence such as a putative WRKY76 encoding transcript, implicated in susceptibility against *Magnoporthe oryzae* but increased tolerance to cold in rice [57] and a putative phenylalanine ammonia-lyase (PAL) encoding transcript. PAL is an important enzyme for salicylic acid production and is a key enzyme in the phenylpropanoid pathway, shown to be induced in response to wounding and fungal infection in *Pinus sylvestris* [58]. A putative map kinase 4 is also down-regulated. In Arabidopsis, map kinase 4 is known to regulate the salicylic acid and jasmonic acid/ ethylene defence signaling [59]. Although it is tempting to speculate that the down-regulation of such important transcripts in defence may, in part, contribute to susceptibility against *F. circinatum*, a detailed time-course of infection in *P. patula* is necessary to determine the full suite of host responses during this susceptible interaction*.*

## Conclusions

This study presents the first transcriptome sequencing and assembly analysis for *Pinus patula*. The *P. patula* v1.0 transcriptome assembly constitutes the largest gene catalogue for this economically important species to date. More than 23 Gb of data was used to assemble 52 112 sequences with a total length of 52 Mb and an average coverage of more than 200×. Of these sequences, 30 844 could be assigned annotations. This transcriptome represents a major genomic resource for future studies on this tropical *Pinus* species, and will be used as the basis for further investigation of the host pathogen interaction between *P. patula* and *F. circinatum*. The workflow used for transcriptome assembly can in future be reapplied and altered as new sequencing data becomes available for *P. patula* to produce a more comprehensive and complete assembly. Furthermore, the workflow implemented during this study could be applied to other species where a high quality genome sequence is not available. One species to which the workflow could be applied in future is *P. tecunumanii*, a species that is closely related to *P. patula* [60] but which displays resistance to *F. circinatum*. Assembly of the *P. tecunumanii* transcriptome would thus allow for further investigation of the mechanisms differentiating resistance and susceptibility through comparison of defence responses in these closely related species.

## Availability of supporting data

The data sets supporting the results of this article are available through the NCBI BioProject repository, [PRJNA301922; http://www.ncbi.nlm.nih.gov/bioproject/301922].

## Additional files

Additional file 1: Table S1. EvidentialGene tr2aacds pipeline output summary. (PDF 138 kb) Additional file 2: Table S2. Assembly statistics for EvidentialGene tr2aacds pipeline merged assembly compared to average statistics for each assembler. (PDF 150 kb) Additional file 3: Table S3. Predicted species distribution for non-pine origin sequences removed from the *Pinus patula* v1.0 transcriptome. (TSV 7 kb) Additional file 4: Figure S1. Molecular function gene ontology distribution for the *Pinus patula* v1.0 transcriptome. (TIF 713 kb) Additional file 5: Table S4. Tribe-MCL gene families and annotations for all 15 species used. (CSV 1210 kb) Additional file 6: Table S5. Conditional reciprocal best BLAST alignment results between full-length Sanger sequenced *Pinus taeda* cDNA and representative *Pinus patula* transcripts for each cDNA. (CSV 20 kb) Additional file 7: Figure S2. Summary statistics for alignment of *Pinus taeda* complete CDS sequences to assembled *Pinus patula* transcripts. Pita = *P. taeda*. The x-axis represents the query *P. taeda* cDNA sequence. The solid y-axis (left) illustrates: cDNA query sequence length (pink circle), *P. patula* subject sequence length (blue square), conditional reciprocal best BLAST alignment length (gold triangle). The dashed y-axis (right) depicts the: percentage identity between sequences (black line), percentage coverage of the *P. taeda* cDNA by the corresponding *P. patula* transcript (green cross) and vice versa (purple plus). (TIF 2325 kb) Additional file 8: Table S6. EBSeq differential expression analysis results comparing expression between inoculated and mock-inoculated data. (TSV 6517 kb) Additional file 9: Table S7. Summarized list of differentially expressed genes between inoculated and mock-inoculated data with annotations. (TSV 68 kb)

## Abbreviations

BUSCO: Benchmarking Universal Single-Copy Orthologs
*ca*: *circa*
CDS: DNA Coding Sequence
CEGMA: core eukaryotic genes mapping approach
CEGs: core eukaryotic genes
CRBB: conditional reciprocal best BLAST
DEPC: diethylpyrolecarbonate
dpi: days post inoculation
EC: enzyme code
GMAP: genomic mapping and alignment program
GO: gene ontology
GSNAP: genomic short-read nucleotide alignment program
NCBI: National Centre for Biotechnology Information
nr: non-redundant
ORF: Open Reading Frame
PCR: polymerase chain reaction
PDA: potato dextrose agar
RBH: reciprocal best hit
RNA-seq: RNA sequencing
USCOs: Universal Single-Copy Orthologs
