# Supplementary material for: Combined de novo and genome guided assembly and annotation of the Pinus patula juvenile shoot transcriptome
Source: BMC Genomics. 2015 Dec 12;16:1057. doi: 10.1186/s12864-015-2277-7 (PMC4676862; doi:10.1186/s12864-015-2277-7)
Supplement: Additional file 1: Table S1. — EvidentialGene tr2aacds pipeline output summary. (PDF 138 kb) [file 12864_2015_2277_MOESM1_ESM.pdf]

Table S1: EvidentialGene tr2aacds pipeline output summary.

Category	Class	Okay	Drop
Primary transcripts with alternatives		41 474	1 849
	a2	5 085	481
Primary transcripts without alternatives		19 451	2 302
	a2	367	24
Alternative transcripts with primary transcripts identified	mid	23 837	1 436
	mid a2	2 069	93
	hi	58 913	27 488
	hi1	79 273	145 823
	hi a2	0	4 603
	mfrag	15 233	1 774
	mfrag a2	1 333	124
Partial alternative transcripts	hi	0	97 926
	hi1	0	63 473
	hi a2	0	9 621
Total		247 035	357 017

a2 = high protein identity.

hi = > 98% sequence identity to primary transcript.

hi1 = very high identity to primary transcript.

mid = lower sequence identity to primary.

mfrag = shorter sequence with lower identity to primary, potential paralog.
