# Supplementary material for: Combined de novo and genome guided assembly and annotation of the Pinus patula juvenile shoot transcriptome
Source: BMC Genomics. 2015 Dec 12;16:1057. doi: 10.1186/s12864-015-2277-7 (PMC4676862; doi:10.1186/s12864-015-2277-7)
Supplement: Additional file 2: Table S2. — Assembly statistics for EvidentialGene tr2aacds pipeline merged assembly compared to average statistics for each assembler. (PDF 150 kb) [file 12864_2015_2277_MOESM2_ESM.pdf]

Table S2: Assembly statistics for EvidentialGene tr2aacds pipeline merged assembly compared to average statistics for each assembler.

Assembly	Merged Assembly				
	Loci	Transcripts	Trinity	Velvet/Oases	SOAPdenovo-Trans
Total Assembled Transcripts	66 377	247 035	134 766	185 574	271 378
Longest Transcript (Kb)	10 839	10 839	12 167	11 490	27 448
Assembly Length (Mb)	63.45	230.62	123.11	216.75	121.16
Mean Sequence Length (nt)	956	934	910	1 195	392
Predicted ORFs in assembly	38 386	134 827	41 425	86 984	33 707
% ORF[1]	85.46%	78.97%	52.70%	55.73%	12.42%
N90	462	480	449	589	356
N70	798	786	728	1 049	983
N50	1 194	1 113	1 122	1 530	1 802
N30	1 692	1 539	1 697	2 158	2 979
N10	2 853	2 514	2 745	3 441	27 448
GC	46.87%	46.00%	42.55%	42.98%	42.91%
CPG ratio[2]	1.71	1.69	1.65	1.65	1.69
% N[3]	0.13%	0.13%	0.00%	0.05%	1.08%

[1] mean percentage of a contig covered by an open reading frame (ORF).

[2] counts of CpG sites relative to the expected number.

[3] proportion of bases in the assembly that are N.
