# Supplementary figures and images for: Combined de novo and genome guided assembly and annotation of the Pinus patula juvenile shoot transcriptome
Source: BMC Genomics. 2015 Dec 12;16:1057. doi: 10.1186/s12864-015-2277-7 (PMC4676862; doi:10.1186/s12864-015-2277-7)

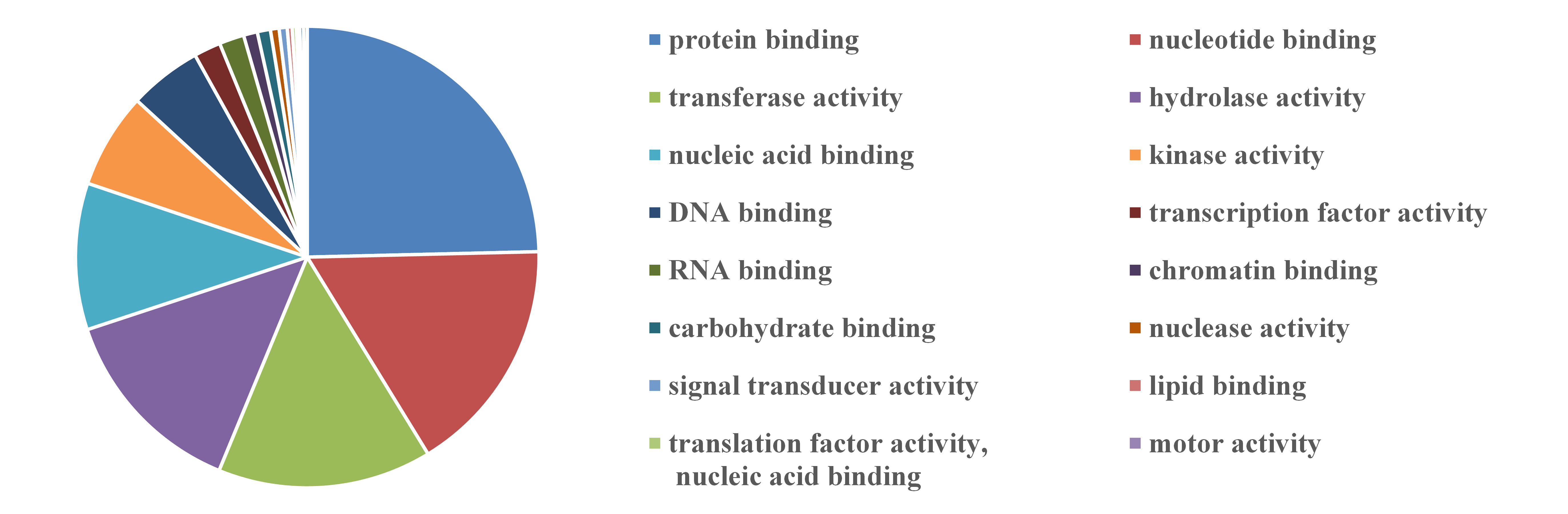

Supplement: Additional file 4: Figure S1. — Molecular function gene ontology distribution for the Pinus patula v1.0 transcriptome. (TIF 713 kb) [file 12864_2015_2277_MOESM4_ESM.tif]

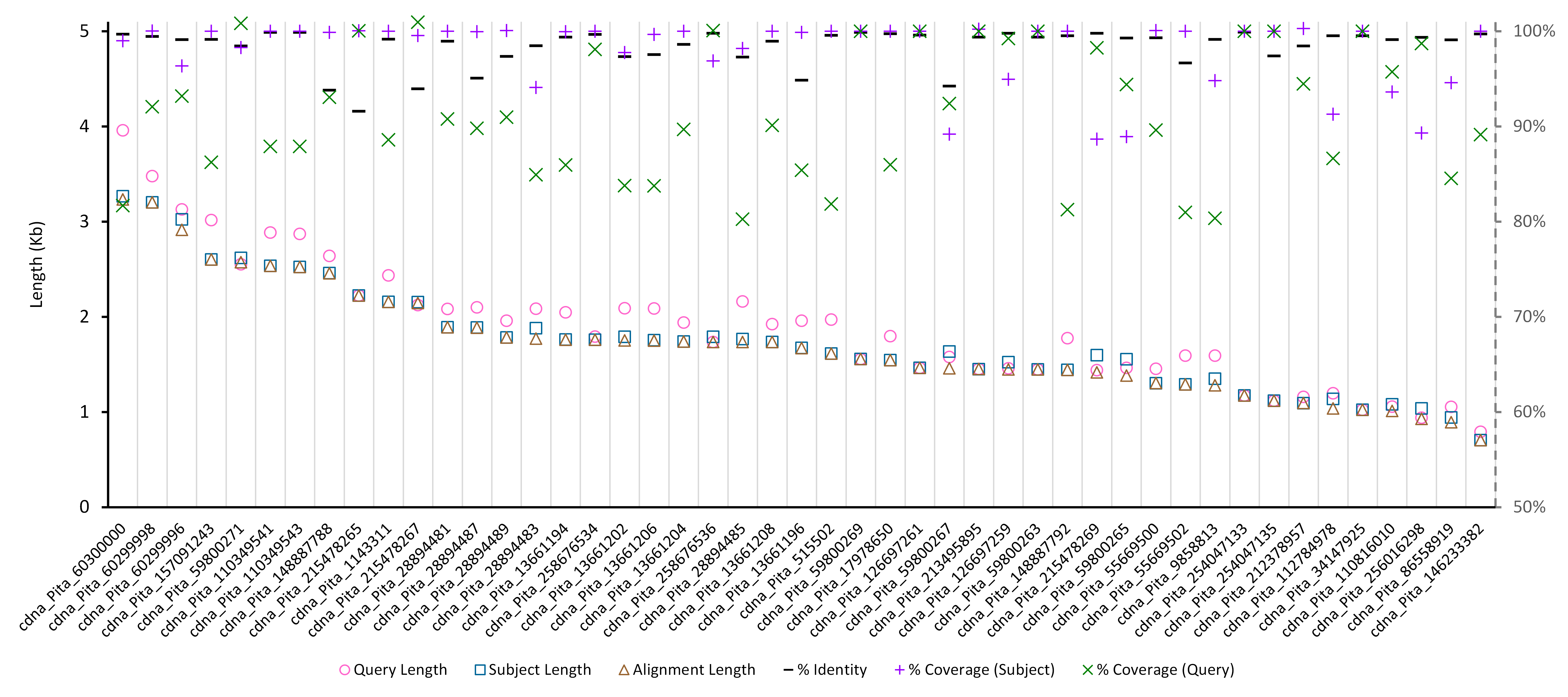

Supplement: Additional file 7: Figure S2. — Summary statistics for alignment of Pinus taeda complete CDS sequences to assembled Pinus patula transcripts. Pita = P. taeda. The x-axis represents the query P. taeda cDNA sequence. The solid y-axis (left) illustrates: cDNA query sequence length (pink circle), P. patula subject sequence length (blue square), conditional reciprocal best BLAST alignment length (gold triangle). The dashed y-axis (right) depicts the: percentage identity between sequences (black line), percentage coverage of the P. taeda cDNA by the corresponding P. patula transcript (green cross) and vice versa (purple plus). (TIF 2325 kb) [file 12864_2015_2277_MOESM7_ESM.tif]
